# Supplementary material for: Can open-defecation free (ODF) communities be sustained? A cross-sectional study in rural Ghana
Source: PLoS One. 2022 Jan 7;17(1):e0261674. doi: 10.1371/journal.pone.0261674 (PMC8740968; doi:10.1371/journal.pone.0261674)
Supplement: S1 Fig — (DOCX) [file pone.0261674.s001.docx]

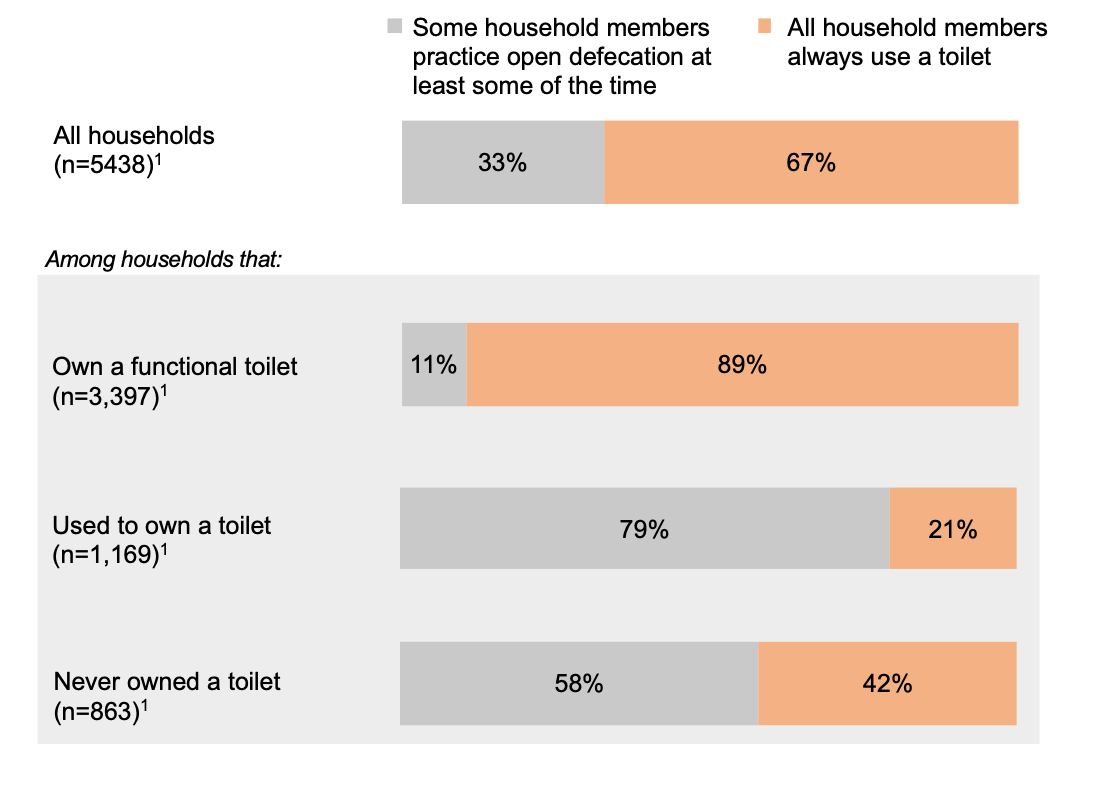


**S1 Fig. Levels of open defecation (“Any OD”) among toilet owners, past owners, and non-owners.**^1^ We were unable to determine the “Any OD” behavior for 177 households.
