# Supplementary material for: Can open-defecation free (ODF) communities be sustained? A cross-sectional study in rural Ghana
Source: PLoS One. 2022 Jan 7;17(1):e0261674. doi: 10.1371/journal.pone.0261674 (PMC8740968; doi:10.1371/journal.pone.0261674)
Supplement: S2 Fig — (DOCX) [file pone.0261674.s002.docx]

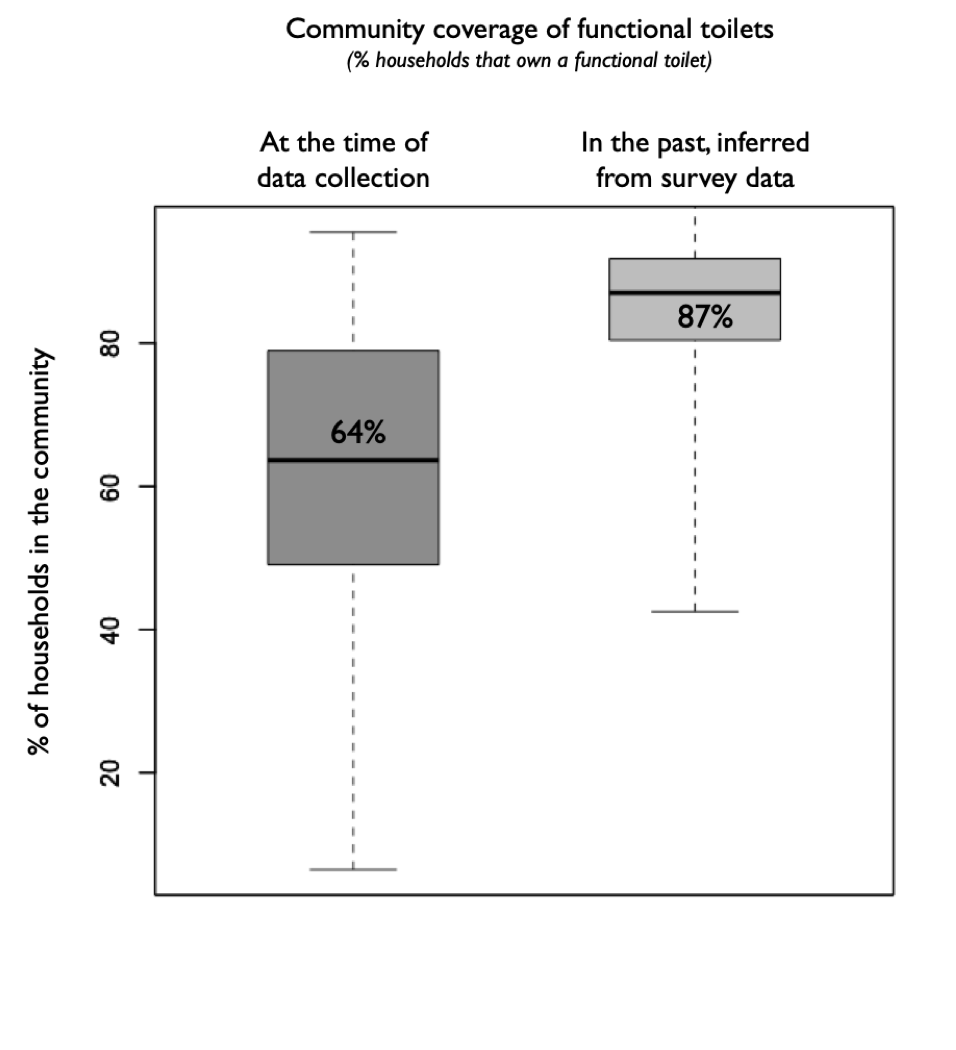


**S2 Fig. Distribution of community-level toilet coverage at the time of data collection and as estimated peak in the past.** To make this peak estimate, we added current toilet owners and households that reported having owned a toilet in the past. It is possible that this is an overestimate, since these groups may not all have owned toilets at the same time. The boxplots display the median, interquartile range, min, and max of the two indicators.
