# Supplementary material for: Can open-defecation free (ODF) communities be sustained? A cross-sectional study in rural Ghana
Source: PLoS One. 2022 Jan 7;17(1):e0261674. doi: 10.1371/journal.pone.0261674 (PMC8740968; doi:10.1371/journal.pone.0261674)
Supplement: S3 Fig — (DOCX) [file pone.0261674.s003.docx]

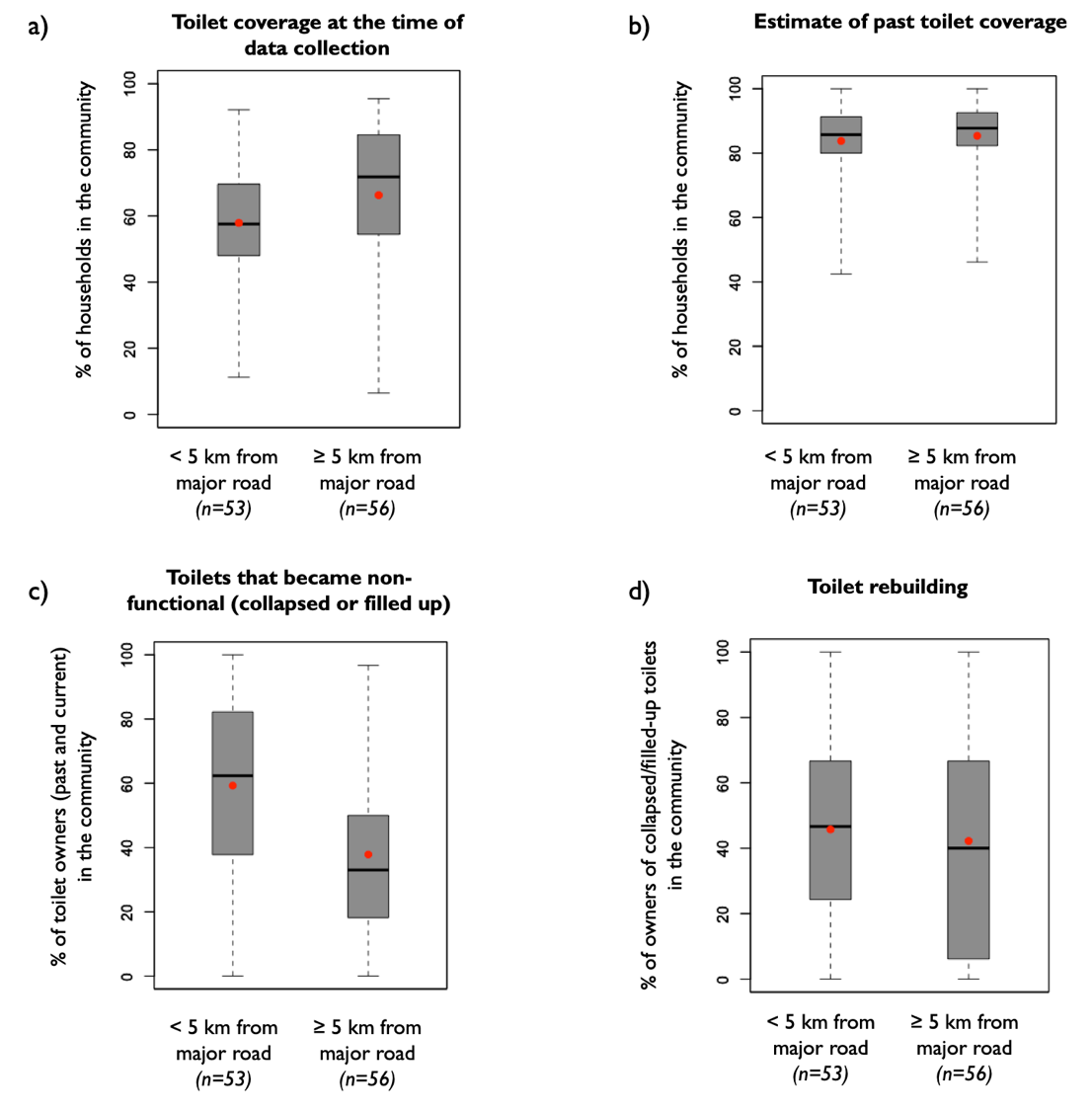


**S3 Fig. Evolution of toilet coverage according to communities’ distance from major roads.** The boxplots display the median, interquartile range, min, and max of community-level statistics. The means are displayed with red dots.
