# Supplementary material for: Can open-defecation free (ODF) communities be sustained? A cross-sectional study in rural Ghana
Source: PLoS One. 2022 Jan 7;17(1):e0261674. doi: 10.1371/journal.pone.0261674 (PMC8740968; doi:10.1371/journal.pone.0261674)
Supplement: S1 Table — (DOCX) [file pone.0261674.s004.docx]

**S1 Table. Definition of community and household characteristics examined in this study.**

| Indicator/variable | Definition | Collection method | Household-level variable | Community-level variable |
| --- | --- | --- | --- | --- |
| **Community characteristics** | | | | |
| Number of households | Number of households living within community boundaries. | Calculated based on number of survey records | - | Continuous |
| Population density^1^ | Average number of people per km^2^ in community. | Computed using georeferenced dataset [19]. | - | Continuous (people/km^2^) |
| Distance to major roads | Approximate distance from community to a major road (as defined in Open Street Maps). | Computed using georeferenced dataset [19]. | - | Continuous (km) |
| Time to city^1^ | Approximate travel time from community to the closest city of 50,000 people or more. | Computed using georeferenced dataset [19]. | - | Continuous (min) |
| Shallow groundwater | Water table in community was less than 15 feet deep. | Reported by chief or elder | - | Binary (yes/no) |
| Sandy soil^1^ | Community had areas with sandy or unstable soil. | Reported by chief or elder | - | Binary (yes/no) |
| Rocky soil | Community had areas with rocky or hard-to-dig soil. | Reported by chief or elder | - | Binary (yes/no) |
| Annual flooding | Community or parts of the community experienced flooding every year. | Reported by chief or elder | - | Binary (yes/no) |
| Nearby waterbody | There was a waterbody (lake, river, pond) within 5 min walk of the community. | Reported by chief or elder | - | Binary (yes/no) |
| Nearby forest^1^ | There was dense vegetation (forest, dense bushed, high grasses) within 5 min walk of the community. | Reported by chief or elder | - | Binary (yes/no) |
| Proportion in lower two wealth quintiles | Proportion of households in the lowest two quintiles (40%) of an asset-wealth index. | Calculated from survey data | - | Continuous  (% households) |
| Proportion with mobile phone^1^ | Proportion of households in community owning a mobile phone. | Calculated from survey data | - | Continuous  (% households) |
| LEAP enrollment | Community was enrolled in the government Livelihood Empowerment Against Poverty (LEAP) program. | Reported by chief or elder | - | Binary (yes/no) |
| Improved water source^1^ | At least one of the main water sources for drinking and cooking in community was improved (piped, borehole, protected dug well, protected spring, rainwater, sachet water). | Reported by chief or elder | - | Binary (yes/no) |
| Water source in community | There was at least one water source located within the community boundaries. | Reported by chief or elder | - | Binary (yes/no) |
| Past water programs^1^ | Community had received NGO programs related to water supply. | Reported by chief or elder | - | Binary (yes/no) |
| Past sanitation programs | Community had received NGO programs related to sanitation (other than CLTS). | Reported by chief or elder | - | Binary (yes/no) |
| Past handwashing programs^1^ | Community had received NGO programs related to handwashing. | Reported by chief or elder | - | Binary (yes/no) |
| VSLA | Community had a Village Savings and Loans Association as a result of past development programs. | Reported by chief or elder | - | Binary (yes/no) |
| Months since ODF verification | Time since community was verified to meet ODF requirements by local government officials. | Reported by UNICEF | - | Continuous (months) |
| Fines for open defecation | Community had a system of fines to punish members found open defecating. | Reported by chief or elder | - | Binary (yes/no) |
| Technical volunteers | UNICEF had trained technical volunteers on toilet construction in community. | Reported by chief or elder | - | Binary (yes/no) |
|  | | | | |
| **Household characteristics** | | | | |
| Household size | Number of persons composing the household, excluding persons who lived there less than 6 months in the year. | Self-reported | Continuous (integer) | - |
| Compound size | Number of households in compound. | Self-reported | Continuous (integer) | - |
| Presence of children under five | Household included one or more children under five years old. | Self-reported | Binary (yes/no) | - |
| Presence of school-age children^2^ | Household included one or more children between five and fourteen years old. | Self-reported | Binary (yes/no) | - |
| Presence of elderly people | Household included one or more person over 65 years old. | Self-reported | Binary (yes/no) | - |
| Age of household head^2^ | Age of the household head. | Self-reported | Continuous (years) | - |
| Household head completed primary education | Household head had at least completed primary school. | Self-reported | Binary (yes/no) | - |
| Wealth index | Asset-wealth index computed across study households according to method of [25]. | Calculated from survey data | Continuous | - |
| LEAP status | At least one household member was a recipient of the government LEAP program. | Self-reported | Binary (yes/no) | - |
| Use of surface water | Surface water is the main source of drinking water for the household. | Self-reported | Binary (yes/no) | - |
| Female household head | The head of household was a woman. | Self-reported | Binary (yes/no) | - |
| Presence of persons with physical/mental challenge or chronic illness | One or more household members were physically or mentally challenged, or had a chronic illness. | Self-reported | Binary (yes/no) | - |
| Household head with physical/mental challenge or chronic illness^2^ | The head of household was physically or mentally challenged, or had a chronic illness. | Self-reported | Binary (yes/no) | - |

^1^ These community characteristics were excluded from multivariate analyses due to collinearity: population density and time to city were collinear to distance to major roads, sandy soil was collinear to nearby waterbody, annual flooding was collinear to nearby forest, proportion owning mobile phone was collinear to proportion in lowest two wealth quintiles, improved water source was collinear to water source in community, past water and handwashing programs were collinear to past sanitation programs.

^2^ These household characteristics were excluded from multivariate analyses due to collinearity: presence of school-age children was collinear to household size, age of household head was collinear with presence of children under five and presence of elderly people, household with challenge/chronic illness was collinear to presence of persons with challenge/chronic illness.
