# Supplementary material for: Can open-defecation free (ODF) communities be sustained? A cross-sectional study in rural Ghana
Source: PLoS One. 2022 Jan 7;17(1):e0261674. doi: 10.1371/journal.pone.0261674 (PMC8740968; doi:10.1371/journal.pone.0261674)
Supplement: S2 Table — (DOCX) [file pone.0261674.s005.docx]

**S2 Table. Characteristics of study communities (n=109).**

|  | Community characteristic | Mean or proportion |
| --- | --- | --- |
| **Demographic** | Mean number of compounds^1^ per community (inter-quartile range) | 31 (18-39) |
|  | Mean number of households^2^ per community (inter-quartile range) | 52 (28-69) |
|  | Mean number of persons per community (inter-quartile range) | 357 (193-460) |
|  | Mean population density (pp/km^2^) (inter-quartile range) | 183 (17-168) |
|  | Mean distance to major road (km) (inter-quartile range) | 6 (2-7) |
|  | Mean travel time to nearest city (minutes) (inter-quartile range) | 73 (45-94) |
| **Environmental** | **Groundwater depth**^3^  5 to 10 feet  10 to 15 feet  More than 15 feet | 3%  24%  73% |
|  | **Soil type**^3^  Community has locations with sandy/unstable soil  Community has locations with rocky soil | 83%  32% |
|  | **Flooding**^3^  Annually  Less than annually  Never | 16%  8%  76% |
|  | Community has nearby waterbody (within 5 min walk from edge of community)^3^ | 41% |
|  | Community has nearby tree cover (within 5 min walk from edge of community)^3^ | 25% |
| **Socioeconomic** | **Top three problems experienced by community**^3^  Water  Education  Road  Poverty  Electricity | 78%  60%  51%  39%  39% |
|  | Mean proportion of households in the lowest two wealth quintiles (min-max and inter-quartile range) | 42% (9-90%, 28-53%) |
|  | Mean proportion of households owning a mobile phone (inter-quartile range) | 70% (61-81%) |
|  | Community is enrolled in LEAP (government poverty alleviation program)^3^ | 58% |
| **Water** | **Main water sources for drinking and cooking**^3^  Piped or other improved  At least one water source is located within the community  All water sources are located more than 10 min away | 77%  50%  30% |
| **Development programs** | **Past WASH programs**^3^  Community has received water interventions  Community has received sanitation interventions (other than CLTS)  Community has received handwashing interventions | 29%  31%  25% |
|  | Community has a Village Savings and Loans Association (VSLA)^3^ | 37% |
| **CLTS history** | Mean time since ODF verification^4^ (months) (min-max and inter-quartile range) | 16 (3-32, 9-22) |
|  | **Sanctions for open defecation**^3^  Community has a system of fines for open defecation  Community has applied fines in the past year | 85%  58% |
|  | Community has volunteers trained on latrine construction (by UNICEF)^3^ | 44% |

^1^ A compound is a plot enclosed by wall and can be composed of several households.

^2^ A household is a group of people living in the same dwelling and eating meals together or recognizing a single head of household.

^3^ These characteristics were reported by community chiefs or elders. Data were missing for groundwater depth (14), past WASH programs (4), VSLA (12), and trained volunteers (1).

^4^ Data were missing for one community.
