# Supplementary material for: Can open-defecation free (ODF) communities be sustained? A cross-sectional study in rural Ghana
Source: PLoS One. 2022 Jan 7;17(1):e0261674. doi: 10.1371/journal.pone.0261674 (PMC8740968; doi:10.1371/journal.pone.0261674)
Supplement: S3 Table — (DOCX) [file pone.0261674.s006.docx]

**S3 Table. Results of multivariate logistic regressions for household-level sanitation conditions.** We present odds ratios (OR), p-values, and clustered standard errors. Statistically significant results (p<0.05) are indicated in bold font on grey background.

|  | Ownership of functional toilet | | | Ownership of functional toilet with full superstructure | | | Any open defecation | | | Primary open defecation | | | Toilet rebuilding | | |
| --- | --- | --- | --- | --- | --- | --- | --- | --- | --- | --- | --- | --- | --- | --- | --- |
|  | OR | p-value | st. err. | OR | p-value | st. err. | OR | p-value | st. err. | OR | p-value | st. err. | OR | p-value | st. err. |
| Household size | **1.17** | **<0.001** | **0.05** | **1.15** | **0.001** | **0.05** | 0.95 | 0.21 | 0.04 | 0.95 | 0.21 | 0.04 | 1.08 | 0.13 | 0.06 |
| Compound size | 0.95 | 0.47 | 0.07 | 1.03 | 0.69 | 0.07 | **1.14** | **0.02** | **0.06** | 1.13 | 0.06 | 0.08 | 1.00 | 0.97 | 0.09 |
| Has children under five | **0.92** | **0.03** | **0.03** | **0.88** | **0.002** | **0.04** | **1.14** | **<0.001** | **0.04** | **1.09** | **0.03** | **0.04** | 0.90 | 0.05 | 0.05 |
| Has elderly person | 0.97 | 0.42 | 0.03 | 0.99 | 0.74 | 0.03 | 1.07 | 0.06 | 0.04 | 1.01 | 0.72 | 0.04 | 0.97 | 0.44 | 0.04 |
| Head completed primary education | **0.91** | **0.02** | **0.04** | 0.96 | 0.37 | 0.04 | **0.91** | **0.01** | **0.03** | 0.93 | 0.11 | 0.04 | 1.00 | 0.97 | 0.06 |
| Wealth index | **1.16** | **0.01** | **0.07** | **1.22** | **<0.001** | **0.07** | 0.88 | 0.06 | 0.06 | **0.84** | **0.03** | **0.07** | **1.44** | **<0.001** | **0.13** |
| LEAP household | **1.13** | **0.05** | **0.07** | 1.10 | 0.10 | 0.06 | 0.91 | 0.16 | 0.06 | 0.87 | 0.08 | 0.07 | 1.11 | 0.22 | 0.10 |
| Uses surface water for drinking | 0.93 | 0.33 | 0.08 | 0.94 | 0.41 | 0.08 | 1.15 | 0.13 | 0.11 | 1.17 | 0.10 | 0.12 | 0.92 | 0.47 | 0.11 |
| Has a female household head | **0.76** | **<0.001** | **0.03** | **0.81** | **<0.001** | **0.03** | 1.05 | 0.19 | 0.04 | 1.04 | 0.32 | 0.05 | 1.00 | 1.00 | 0.06 |
| Has a challenged or chronically ill person | 0.95 | 0.10 | 0.03 | **0.94** | **0.03** | **0.03** | **1.20** | **<0.001** | **0.05** | **1.09** | **0.05** | **0.05** | 1.00 | 0.98 | 0.05 |
| Months since ODF verification (community) | **0.69** | **<0.001** | **0.06** | **0.65** | **<0.001** | **0.05** | **1.34** | **0.01** | **0.15** | **1.40** | **0.004** | **0.17** | 0.81 | 0.13 | 0.12 |
