# Supplementary material for: Can open-defecation free (ODF) communities be sustained? A cross-sectional study in rural Ghana
Source: PLoS One. 2022 Jan 7;17(1):e0261674. doi: 10.1371/journal.pone.0261674 (PMC8740968; doi:10.1371/journal.pone.0261674)
Supplement: S5 Table — (DOCX) [file pone.0261674.s008.docx]

**S5 Table. Results of multivariate beta regressions for community-level sanitation conditions with a different model.** Compared to the model presented in Table S4, we have replaced Annual Flooding and Nearby Waterbody with their collinear variables: Nearby Forest and Sandy Soil, respectively (red). Statistically significant results (p<0.05) are indicated in bold font on grey background.

|  | Community coverage of functional toilets  *(% households that own a functional toilet)* | | | Community prevalence of open defecation  *(% households that usually practice)* | | |
| --- | --- | --- | --- | --- | --- | --- |
|  | **coeff** | **p-value** | **st. err.** | **coeff** | **p-value** | **st. err.** |
| Number of households | -0.03 | 0.75 | 0.09 | 0.07 | 0.57 | 0.11 |
| Distance to major road | **0.22** | **0.05** | **0.11** | -0.01 | 0.93 | 0.14 |
| Shallow groundwater (<15 feet) | 0.18 | 0.07 | 0.10 | -0.17 | 0.19 | 0.13 |
| Rocky soil | **-0.27** | **0.001** | **0.08** | 0.20 | 0.07 | 0.11 |
| Sandy soil | -0.16 | 0.07 | 0.09 | 0.01 | 0.94 | 0.12 |
| Nearby forest | -0.11 | 0.25 | 0.10 | 0.17 | 0.17 | 0.12 |
| Population in lower two wealth quintiles | -0.06 | 0.50 | 0.09 | 0.02 | 0.86 | 0.12 |
| LEAP enrollment | 0.16 | 0.06 | 0.09 | -0.05 | 0.67 | 0.11 |
| Water source in community | -0.07 | 0.37 | 0.08 | 0.03 | 0.77 | 0.11 |
| Past sanitation programs | -0.02 | 0.85 | 0.09 | -0.04 | 0.72 | 0.11 |
| VSLA | 0.11 | 0.24 | 0.09 | -0.23 | 0.06 | 0.12 |
| Months since ODF verification | **-0.35** | **<0.001** | **0.10** | **0.41** | **0.001** | **0.13** |
| Fine for open defecation | **0.29** | **<0.001** | **0.08** | **-0.20** | **0.05** | **0.10** |
| Technical volunteers | -0.01 | 0.89 | 0.09 | 0.04 | 0.72 | 0.12 |
