# Supplementary material for: Can open-defecation free (ODF) communities be sustained? A cross-sectional study in rural Ghana
Source: PLoS One. 2022 Jan 7;17(1):e0261674. doi: 10.1371/journal.pone.0261674 (PMC8740968; doi:10.1371/journal.pone.0261674)
Supplement: S1 Text — (DOCX) [file pone.0261674.s009.docx]

**WASHPaLS Research on the Impact of Targeted Subsidies within ODF Communities**

**Household survey**

| Geographic location | District: |  |
| --- | --- | --- |
|  | Town/Village: |  |
|  | Household ID |  |

| **No.** | **Introduction and demographics** | **Answer Choices** | **Code** | **Logic** |
| --- | --- | --- | --- | --- |
| A1 | Is someone home and available to be interviewed who lives here and is ≥18 years old? | Yes  No | 1  0 | >>A_3 |
| A2 | READ CONSENT FORM  Are you willing to participate in the study? | Yes, available now  Yes, though at a later time  No | 1  2  0 | >>A_6  >>A_3  >>End |
| A3 | How many times have you visited this household? | _________ |  | >>End if >2 |
| A4 | May a household member who is ≥18 years old be available at a later time? | Yes  No | 1  0 | >>A_6  >>End |
| A5 | When would be a good time to return? | _____________ |  |  |
| A6a | Family name/last name: | |  |  |
| A6b | Respondent first name: | |  |  |
| A6c | Popular name: | |  |  |
| A7 | Respondent gender: | Female  Male | 2  1 |  |
| Now I am going to ask you questions about the composition of your household. | | | | |
| A8 | Are you the head of household?  *We are asking about head of HOUSEHOLD, not head of FAMILY* | Yes  No | 1  0 |  |
| A9 | What is your age?  *(Ask birth year if doesn’t know)* | ___________ years |  | >>End if <18 |
| A10 | What is YOUR highest education level? | None  Kindergarten  Primary school  Junior high school/Middle school  Senior high school/Secondary  Vocational/technical training (e.g. tailoring, masonry)  Teacher training/nursing  Post secondary diploma/HND  Bachelor degree  Post graduate  Other: _______________  Refuse to answer  Don’t know | 0  1  2  3  5  6  7  8  9  10  96  98  99 |  |
| A11 | What is YOUR marital status? | Married  Living together  Separated  Divorced  Never married/single  Widowed  Other: __________ | 1  2  3  4  5  6  96 |  |
| A12 | Do YOU have children under 15 under your care? | Yes  No  Refuse to answer | 1  0  98 |  |
| A13 | What is YOUR main occupation?  *In the last 12 months* | Agriculture  Selling produce or goods  Cooperatives  Private Sector Informal  Private Sector Formal  Government sector  NGOs (local & International)  No occupation, stay home  Other:__________  Refuse to answer  Don’t know | 1  2  3  4  5  6  7  0  96  98  99 |  |
| A14 | *[IF A8=0]*  First name of head of household: |  |  |  |
| A15 | *[IF A8=0]*  What is the gender of the head of household? | Female  Male | 2  1 |  |
| A16 | *[IF A8=0]*  What is the age of the head of household?  *(Ask birth year if doesn’t know)* | ______________ years |  |  |
| A17 | *[IF A8=0]*  What is the highest education level of the head of household? | None  Kindergarten  Primary school  Junior high school/Middle school  Senior high school/Secondary  Vocational/technical training (e.g. tailoring, masonry)  Teacher training/nursing  Post secondary diploma/HND  Bachelor degree  Post graduate  Other: _______________  Refuse to answer  Don’t know | 0  1  2  3  5  6  7  9  10  11  96  98  99 |  |
| A18 | *[IF A8=0]*  What is the marital status of the head of household? | Married  Living together  Separated  Divorced  Never married/single  Widowed  Other: __________ | 1  2  3  4  5  6  96 |  |
| A19 | *[IF A8=0]*  Does the head of household have children under 15 under his/her care? | Yes  No  Refuse to answer | 1  0  98 |  |
| A20 | *[IF A8=0]*  What is the main occupation of the head of household?  *In the last 12 months* | Agriculture  Selling produce or goods  Cooperatives  Private Sector Informal  Private Sector Formal  Government sector  NGOs (local & International)  No occupation, stay home  Other:__________  Refuse to answer  Don’t know | 1  2  3  4  5  6  7  0  96  98  99 |  |
| A21 | How many total households live in this house/compound, including your household?  *Count your own household.* | | _______ |  |
| A22 | How many people are in your household, including yourself?  *People who eat and sleep here more than 50% of the time or 6 months in the year).*  *(Probe for children and elders. 99 if doesn’t know)* | | _______ |  |
| A23 | Does your household have a person who is 65 or older? | Yes  Respondent doesn’t know but probably  No  Don’t know | 1  2  0  99 |  |
| A24 | Does your household have a person who is physically or mentally challenged? | Yes  No  Don’t know | 1  0  99 | >>A35  >>A35 |
| A25 | How many household members are physically or mentally challenged? | ___________ |  |  |
| A26 | *[FOR EACH PERSON]:*  Is this person the respondent? | Yes  No  Don’t know | 1  0  99 |  |
| A27 | *[FOR EACH PERSON]:*  *[IF A8=0]*  Is this person the head of household? | Yes  No  Don’t know | 1  0  99 |  |
| A28 | *[FOR EACH PERSON]:*  Is this person a child under 15 years old? | Yes  No  Don’t know | 1  0  99 |  |
| A29 | *[FOR EACH PERSON]:*  What kind of physical/mental challenge(s) does this person suffer from?  *Select all that apply.* | Sight  Hearing  Speech  Physical  Intellect  Other: _________  Refuse to answer  Don’t know | 1  2  3  4  5  96  98  99 |  |
| A30 | Does your household have a person who is chronically ill?  *Any chronic or recurring illness (e.g., HIV, epilepsy, asthma, hepatitis B, diabetes, stroke). But do not count chronic pain.* | Yes  No  Don’t know | 1  0  99 | >>A41  >>A41 |
| A31 | How many household members are chronically ill? | _____________ |  |  |
| A32 | *[FOR EACH PERSON]*  Is this person the respondent? | Yes  No  Don’t know | 1  0  99 |  |
| A33 | *[FOR EACH PERSON]*  *[IF A8=0]*  Is this person the head of household? | Yes  No  Don’t know | 1  0  99 |  |
| A34 | *[FOR EACH PERSON]*  Is this person a child under 15? | Yes  No  Don’t know | 1  0  99 |  |
| A35 | *[FOR EACH PERSON]*  What chronic illness(es) does this person suffer from?  *Select all that apply.* | HIV  Epilepsy  Asthma  Hepatitis B  Diabetes  Hypertension  Cancer  Stroke  Other: __________  Refuse to answer  Don’t know | 1  2  3  4  5  6  7  8  96  98  99 |  |
| A36 | In this household, is there a child under 15 missing a parent? | Yes: child missing one parent  Yes: child missing two parents  No | 1  2  0 |  |
| A37 | Does your household have a widow(er)? | Yes  No  Don’t know | 1  0  99 |  |
| A41 | Has anyone in this household had diarrhea or dysentry in the past 1 week?  *Do not count today.*  *Probe: 3 or more loose stools per day?* | Yes  No  Don’t know | 1  0  99 | >>B1  >>B1 |
| A42 | How many household members have had diarrhea or dysentry in the past 1 week?  *Probe: 3 or more loose stools per day?* | ______________ |  |  |
| A43 | *[For each person]*  What is the age of this person? | ______________ |  |  |

| **No.** | **Toilet ownership** | **Answer Choices** | **Code** | **Logic** |
| --- | --- | --- | --- | --- |
| Now I will ask you questions about your household’s defecation behaviors. | | | | |
| B1 | Where do members of your household usually defecate?  *Refrain from using word “toilet” in the question.* | Flush / Pour flush  Dry pit latrine  Dig & bury, bush, refuse dump, water body  Other: _____________  Don’t know | 2  1  0  96  99 | >>B3  >>B3  >>B3 |
| B2 | Does your household own the toilet facility that you use?  *Owning = contributed to its construction* | Yes, single owner  Yes, co-owner  No  Don’t Know | 1  2  0  99 | >>B5 |
| B10 | *[If B2=0]:*  Who owns the toilet that your household uses?  *Select all that apply.* | Another household IN my house/compound  A household OUTSIDE my house/compound  School  Public latrine  Other: _________  Don’t know | 1  2  3  4  96  99 |  |
| B11a | *[If B1=1 or 2]:*  Do other households in your house/compound also use this toilet?  *Regular users; not passers-by or visitors.* | Yes  No  Don’t know | 1  0  99 |  |
| B11b | *[If B1=1 or 2]:*  Do other households outside your house/compound also use this toilet?  *Regular users; not passers-by or visitors.* | Yes  No  Don’t know | 1  0  99 |  |
| B12 | *[If B1=1 or 2] and [If B11a or B11b =1]:*  Including your own household, how many households use this toilet?  *For more than 10 households, type 10* | _______  Don’t know | 99 |  |
| B3 | Does your household own any toilet facility?  *Owning = contributed to its construction* | Yes-It is usable  Yes, BUT it is not usable  No  Don’t know | 1  2  0  99 | >>B5  >>B13  >>B13 |
| B4 | Why is the toilet facility not usable?  *Select all that apply* | The pit/slab collapsed  The pit got full  The superstructure was destroyed or damaged  Too far  Moved house  The toilet is in construction  Other: _____________  Don’t Know | 1  2  3  4  5  6  96  99 |  |
| B16a | Does any household in your house/compound use this toilet (that you own)?  *Regular users; not passers-by or visitors.* | Yes  No  Don’t know | 1  0  99 |  |
| B16b | Does any household outside your house/compound use this toilet (that you own)?  *Regular users; not passers-by or visitors.* | Yes  No  Don’t know | 1  0  99 |  |
| B17 | *[If B16a or B16b =1]*  How many total households use this toilet (including your household, if applicable)?  *For more than 10 households, type 10. Type 99 if don't know.* |  |  |  |
| B5 | Who constructed your toilet?  *Select all that apply* | Me/my household  Other family members  Neighbors  Paid labor  Communal labor  Help from NGO  Landlord  Other: _____________  Don’t know | 1  2  3  ~~4~~  5  6  7  96  99 |  |
| B6 | How many years ago was your toilet constructed?  *Round number of years. Type 99 if don’t know.* | | |  |
| B7 | Have you made improvements to your toilet since its original construction?  *Renovations count.* | Yes  No  Don’t know | 1  0  99 |  |
| B8 | Is this the first toilet that was ever built by this household? | Yes  No, it is the second toilet  No, it is at least the third toilet  Other: _____________  Don’t know | 1  2  3  96  99 | >>B13  >>B13  >>B13 |
| B9 | How many years ago was your previous toilet constructed?  *Round number of years. Type 99 if don’t know.* | | |  |
| B13 | Have you ever considered constructing a toilet? | Yes, I built one in the past  Yes, I am currently building one  Yes, but I never built one  No  Don’t know | 1  2  3  0  99 | >>B15  >>B15  >>B15  >>B15 |
| B14 | Why are you not using this toilet anymore?  *Select all that apply.* | The pit/slab collapsed  The pit got full  The superstructure was destroyed  Too far  Moved house  Other: __________  Don’t know | 1  2  3  4  5  96  99 |  |
| B15 | What has prevented you from building/rebuilding a toilet?  *Select all that apply.* | Too expensive/not enough money  Too time intensive  Competing priorities  Don’t know how to  Don’t like any of the options  Don’t own my house  Waiting to build a new house  I am too sick to plan it  Rainy or farming season  Not enough space to build one  Other: _____________  Don’t know | 1  2  3  4  5  6  7  8  9  10  96  99 |  |

| **No.** | **Toilet use** | **Answer Choices** | **Code** | **Logic** |
| --- | --- | --- | --- | --- |
| C1 | How often do you personally use the toilet to urinate when at home? | Always  Mostly  Sometimes  Never  Don’t know | 3  2  1  0  99 |  |
| C2a | How often do you personally use the toilet to defecate when at home? | Always  Mostly  Sometimes  Never  Don’t know | 3  2  1  0  99 |  |
| C2b | *[If B1=0]*  How often do you personally use the bush to defecate when at home? | Always  Mostly  Sometimes  Never  Don’t know | 0  1  2  3  99 |  |
| C3a | How often do other adults (above 15 years old) in your household use the toilet to defecate when at home? | Always  Mostly  Sometimes  Never  Don’t know  Not applicable (no other adults) | 3  2  1  0  99  97 |  |
| C3b | *[If B1=0]*  How often do other adults (above 15 years old) in your household use the bush to defecate when at home? | Always  Mostly  Sometimes  Never  Don’t know  Not applicable (no other adults) | 0  1  2  3  99  97 |  |
| C4a | How often do school age children (5-14 years old) in your household use the toilet to defecate when at home? | Always  Mostly  Sometimes  Never  Don’t know  Not applicable (no school age children) | 3  2  1  0  99  97 |  |
| C4b | *[If B1=0]*  How often do school age children (5-14 years old) in your household use the bush to defecate when at home? | Always  Mostly  Sometimes  Never  Don’t know  Not applicable (no school age children) | 0  1  2  3  99  97 |  |
| C5 | *[if C2!=3 or C3!=3 or C4!=3]*  Why do you/members of your household not always use a toilet to defecate when at home?  *Select all that apply.*  *1 and 99 are not compatible with 2-6.* | Doesn’t own toilet  Doesn’t have access to neighbor’s toilet  Not allowed to use toilet  Toilet is not usable (collapsed, full pit, damaged superstructure)  Toilet is not comfortable  Fear that pit/slab will collapse  Security concerns  Toilet lacks privacy  Toilet is dirty  Bad smell  Toilet is too far  Fear of using toilet at night  Prefer open defecation  Pit floods during rainy season  Too many people use the same toilet  Same toilet for women and men  Burden of using water  Mobility issue  Other: _____________  Don’t know | 1  2  3  4  5  6  7  8  9  10  11  12  13  14  15  16  17  18  96  99 |  |
| C6 | Where do small children (0-4 years old) in your household usually defecate?  *Select all that apply.* | Not applicable (no pre-school children)  On the ground/bush/dig & bury/refuse dump/water body  On the ground and mother scoops up  Chamber pot/ potty/container  Napkin/diapers  Mother helps child go to latrine  Other: ___________  Don’t know  Refuse to answer | 97  0  1  2  3  4  96  99  98 | >>C8  >>C8  >>C8  >>C8  >>C8  >>C8 |
| C7 | Where does the mother dispose of the child feces?  *Select all that apply.* | Dig & bury, bush, refuse dump, water body  In latrine  Other: ___________  Don’t know  Refuse to answer | 0  1  96  99  98 |  |
| C8 | You mentioned earlier that your household has (a) physically/mentally challenged person(s).  Where does/do this/these person(s) usually defecate?  *Select all that apply.* | Dig & bury, bush, refuse dump, ground, water body  In bucket/container emptied in bush  In bucket/container emptied in toilet  In toilet  Other: __________  Don’t know | 0  1  2  3  96  99 |  |
| C9 | You mentioned earlier that your household has (a) chronically ill person(s).  Where does/do this/these person(s) usually defecate?  *Select all that apply.* | Dig & bury, bush, refuse dump, ground, water body  In bucket/container emptied in bush  In bucket/container emptied in toilet  In toilet  Other: __________  Don’t know | 0  1  2  3  96  99 |  |
| C10 | You mentioned earlier that your household has (or may have) (an) elderly person(s).  Where does/do this/these person(s) usually defecate?  *Select all that apply.* | Dig & bury, bush, refuse dump, ground, water body  In bucket/container emptied in bush  In bucket/container emptied in toilet  In toilet  Other: __________  Don’t know | 0  1  2  3  96  99 |  |

| **No.** | **Other questions about respondent** | **Answer Choices** | **Code** | **Logic** |
| --- | --- | --- | --- | --- |
| D1 | *[only if uses toilet]*  Did YOU use the toilet for defecation yesterday? | Yes  No  Don’t know/Refuse to answer | 1  0  98 |  |
| D2 | *[only if uses toilet]*  Did YOU use the toilet for defecation the day before yesterday? | Yes  No  Don’t know/Refuse to answer | 1  0  98 |  |
| D3 | *[only if uses toilet]*  Think about the last 7 days. On how many days did you defecate in the open? | No (0) days  Some (1-2-3) days  Most (4-5-6) days  Every day (7)  Refuse to answer | 0  1  2  3  98 |  |

| **No.** | **Toilet observation** | **Answer Choices** | **Code** | **Logic** |
| --- | --- | --- | --- | --- |
| E0 | *[Only if they own a toilet]*  Can you please show me your toilet facility? | Yes  No, it has collapsed  No, no permission  No, other: _____________ | 1  2  3  96 | >> F1  >> F1  >> F1 |
| E1a | *ENUMERATOR: Have you already observed this toilet when surveying a previous household?* | Yes  No | 1  0 | >>E2 |
| E1b | *ENUMERATOR: On what day did you observe this toilet?* | Today  Yesterday  Other: __________ | 1  2  96 |  |
| E1c | *ENUMERATOR: Which household was it on that day?* | 1^st^ HH  2^nd^ HH  3^rd^ HH  4^th^ HH  5^th^ HH  6^th^ HH  7^th^ HH  8^th^ HH  9^th^ HH  10^th^ HH  Other: __________  Don’t know | 1  2  3  4  5  6  7  8  9  10  96  99 | >>F1  >>F1  >>F1  >>F1  >>F1  >>F1  >>F1  >>F1  >>F1  >>F1  >>F1  >>F1 |
| E2 | OBSERVE the toilet | Is there a pit?  Is there some form of superstructure?  Is the pit/slab collapsed?  Is the pit full? | Y/N/dk  Y/N/dk  Y/N/dk  Y/N/dk |  |
| E3 | OBSERVE: What kind of toilet facility does the household own? | Flush / Pour flush to pit  VIP/Single pit with concrete slab  KVIP with concrete slab  Pit with traditional slab  Open pit without slab  Other: _____________  Cannot observe | 1  2  3  4  5  96  99 |  |
| E4 | OBSERVE: What is the main material of the latrine floor? | Poured concrete  Pre-cast concrete  Wood+ Packed mud + Cement plastering  Wood+ Packed mud + Cow dung plastering  Wood + Packed mud  Packed mud only  Wood only  Plastic  Other: _____________  Cannot observe | 1  2  3  4  5  6  7  8  96  99 |  |
| E5 | OBSERVE: How many walls does the latrine have?  *[Walls can be of any materials]* | Four walls or round walls (full height)  Less than four walls  Partly collapsed walls  No walls | 1  2  3  0 | >>E7 |
| E6 | OBSERVE: What is the main material of the latrine walls? | Concrete blocks  Bricks  Stone + packed mud  Wood/bamboo + packed mud  Packed mud + cement plastering  Packed mud + cow dung plastering  Packed mud only  Wood  Plastic  Bamboo/thatch  Zinc  Other: _____________  Cannot observe | 1  2  3  4  5  6  7  8  9  10  11  96  99 |  |
| E7 | OBSERVE: What is the main material of the latrine roof? | Concrete  Clay tiles  Wood  Thatch/grass  Bamboo rods  Plastic  Zinc  No roof  Other: _____________  Cannot observe | 1  2  3  4  5  6  7  0  96  99 |  |
| E8 | OBSERVE: Is there a curved wall, door, or curtain for privacy? | Yes  No  Cannot observe | 1  0  99 | >>E10  >>E10 |
| E9 | OBSERVE: Can the door be locked from the inside? | Yes  No  Cannot observe | 1  0  99 |  |
| E10 | OBSERVE: Does the toilet have any of the following: | A raised seat  Support handles  Stairs/ steps  Ventilation pipe exiting the latrine | Y/N/dk  Y/N/dk  Y/N/dk  Y/N/dk |  |
| E11 | OBSERVE: Does the pit have a well-fitting lid or covering?  *Whether or not the lid is closed at the time of the survey.* | Yes  Yes, BUT not well-fitting or not closed  No  Cannot observe | 2  1  0  99 |  |
| E12 | OBSERVE: Does the toilet smell? | Yes, very badly  Yes, some  Yes, very little  No  Cannot observe | 3  2  1  0  99 | >>E14  >>E14 |
| E13 | OBSERVE: Are there feces inside the pit? | Yes  No  Cannot observe | 1  0  99 | If E3!=1 |
| E14 | OBSERVE: What anal cleansing material is present (used or unused)?  *Select all that apply. 0 and 99 not compatible with other options.* | Water  Toilet paper  Maize cobs  Newspaper/other paper  Leaves  Sticks  Cloth  Other: _____________  None present  Cannot observe | 1  2  3  4  5  6  7  96  0  99 |  |
| E15 | OBSERVE: Is there water available for flushing? | Yes  No  Cannot observe | 1  0  99 | only if E3=1 |
| E16 | OBSERVE: Is the slab wet? | Yes  No  Cannot observe | 1  0  99 | only if E3=1 |
| E17 | OBSERVE: Is the path to the toilet walked on? | Yes  No  Cannot observe | 1  0  99 |  |
| E18 | OBSERVE: Are there flies present in the facility? | Yes  No  Cannot observe | 1  0  99 |  |
| E19 | OBSERVE: Is there a handwashing facility near the latrine? | Yes  Yes, BUT broken  No  Cannot observe | 1  2  0  99 | >>E21  >>E21 |
| E20 | OBSERVE: Is water present for handwashing? | Yes  No  Cannot observe | 1  0  99 |  |
| E21 | OBSERVE: Is soap and/or ash present for handwashing? | Yes  No  Cannot observe | 1  0  99 |  |
| E22 | What type of pit lining does your toilet have? | Sealed concrete tank  Lined pit (blocks)  Lined pit (precast concrete ring)  Lined pit (wood)  Lined pit (plastic)  Mud+ Cement plastering  Unlined pit  Other: _____________  Don’t know | 1  2  3  4  5  6  0  96  99 |  |
| E23 | *[If E14=0]*  What does your household use for anal cleansing? | Water  Toilet paper  Maize cobs  Newspaper/other paper  Leaves  Sticks  Cloth  Other: _____________  Don’t know | 1  2  3  4  5  6  7  96  99 |  |

| **No.** | **Satisfaction with toilet** | **Answer Choices** | **Code** | **Logic** |
| --- | --- | --- | --- | --- |
| F1 | *[only if uses or owns toilet]*  This question is about the toilet that you (sometimes) use. Are you satisfied with this toilet as a place to defecate?  *[the latrine they use, whether they own it or not]. Use visual scale.* | Very satisfied  Satisfied  Somewhat NOT satisfied  Very NOT satisfied  Refuse to answer  Don’t know | 4  3  2  1  98  99 | >>G1  >>G1  >>G1 |
| F2 | Why are you not satisfied with your toilet as a place to defecate?  *Select all that apply.* | Dirty  Fear of infection  Inconveniently located  Security concerns  Fear of going at night  Toilet is not comfortable  I fear the pit/slab will collapse  Dark  Not private  Bad smell  Too many bugs/flies  Too small, no space  Pit is full  Pit floods during rainy season  Taboo to defecate over another’s man feces  Too many people use the same toilet  Same toilet for men and women  Wants to upgrade roof  Wants to upgrade walls  Wants to upgrade floor  No door  No ventilation  Burden of using water  Other: _____________  Don’t know | 1  2  3  4  5  6  7  8  9  10  11  12  13  14  15  16  17  18  19  20  21  22  23  96  99 |  |

| **No.** | **Socioeconomic indicators** | **Answer Choices** | **Code** | **Logic** |
| --- | --- | --- | --- | --- |
| Now I will ask you questions about your household dwelling and assets. | | | | |
| G1 | How many rooms does your household occupy for living, eating, and sleeping?  *Do not count bathroom, kitchen, storage rooms, or toilet.* |  |  |  |
| G2 | What is the main source of lighting for your dwelling? | Electricity (mains)  Electricity (private generator)  Kerosene lamp  Gas lamp  Solar energy  Candle  Flashlight/Torch  Firewood  Crop residue  None  Other: __________  Refuse to answer  Don’t know | 1  2  3  4  5  6  7  8  9  0  96  98  99 |  |
| G3 | What is the main construction material used for the dwelling’s outer wall? | Mud/Mud bricks/Earth  Wood  Metal Sheet/ Slate/Asbestos  Stone  Burnt bricks  Cement blocks/Concrete  Landcrete  Bamboo  Palm leaves/ Thatch/Grass  Other: ___________  Cannot observe/don’t know | 1  2  3  4  5  6  7  8  9  96  99 |  |
| G4 | What is the main material used for the dwelling’s roof? | Mud/Mud bricks/Earth  Wood  Zinc  Slate/Asbestos  Cement/Concrete  Bamboo  Palm leaves/Thatch/Grass  Roofing tile  Other: ____________  Cannot observe/don’t know | 1  2  3  4  5  6  7  8  96  99 |  |
| G5 | What is the main construction material used for the dwelling’s floor? | Earth/Mud  Cement/Concrete  Stone  Burnt brick  Wood  Vinyl tiles  Ceramic/Porcelain/Granite/Marble tiles  Terrazzo/Terrazzo tiles  Other: __________________  Cannot observe/don’t know | 1  2  3  4  5  6  7  8  96  99 |  |
| G6 | What is the main source of drinking water for members of your household? | Piped water inside dwelling  Piped water to plot  Piped water to neighbor  Piped water to public tap/standpipe  Tube-well or borehole  Protected dug well  Unprotected dug well  Protected spring  Unprotected spring  Rainwater  Tanker truck/vendor  Bottled water  Sachet water  River/stream  Dugout/pond/lake/dam/canal  Other: ________  Refuse to answer  Don’t know | 1  2  3  4  5  6  7  8  9  10  11  12  13  14  15  96  98  99 |  |
| G7 | Does your household own livestock?  *Probe for bullocks.*  *Enter number. If more than 8, enter the number ‘8’. If unknown, enter ‘99’.* | Donkey, horse, bullock  Cattle (cows AND calves)  Sheep  Goats  Pigs  Poultry, guinea fowl | __  __  __  __  __  __ |  |
| G8 | Does your household own the following items related to agriculture and fishing? | Tractor/harvester  Plough  Trailer/cart  Spraying machine  Canoe  Fishing net  Food processor/blender | Y/N/dk  Y/N/dk  Y/N/dk  Y/N/dk  Y/N/dk  Y/N/dk  Y/N/dk |  |
| G9 | Does anyone in your household own any of the following items? | Sewing machine  Stove (kerosene, gas, electric)  Refrigerator/freezer  Box or electric iron  Radio/Radio cassette/CD player  Television  Computer/tablet  Fan  Car  Motorbike/Tricycle  Bicycle  Mobile phone  Wall clock  Bed  Table  Chair  Cabinet/cupboard  Wrist watch | Y/N/dk  Y/N/dk  Y/N/dk  Y/N/dk  Y/N/dk  Y/N/dk  Y/N/dk  Y/N/dk  Y/N/dk  Y/N/dk  Y/N/dk  Y/N/dk  Y/N/dk  Y/N/dk  Y/N/dk  Y/N/dk  Y/N/dk  Y/N/dk |  |
| G10 | Does your household own land for farming/pastoralism? | Yes  No  Refuse to answer  Don’t know | 1  0  98  99 | >>G16  >>G16  >>G16 |
| G11 | How many acres does your household own for farming/pastoralism?  *Type 99 if don’t know* | _______________ |  |  |
| G12 | Does your household have access to land for farming/pastoralism? | Yes  No  Refuse to answer  Don’t know | 1  0  98  99 | >>G16  >>G16  >>G16 |
| G13 | How many acres does your household have access to for farming/pastoralism?  *Type 99 if don’t know* | ________________ |  |  |
| G14a | *If household has a mobile phone:*  Phone number:  *If don’t know: use *156# for MTN and *127# for Vodafone.* | MTN: ____________ |  |  |
| G14b |  | Vodafone: ______________ |  |  |
| A38 | Does someone in your household have a LEAP card?  *(Ask to see the LEAP card)* | Yes  No  Don’t know | 1  0  99 |  |
| A39 | *[IF A43=1]*  Does the respondent have a LEAP card? | Yes  No  Don’t know | 1  0  99 |  |
| A40 | *[IF A8=0 AND A43=1]*  Does the head of household have a LEAP card?  *Consider asking to see the LEAP card if needed to confirm.* | Yes  No  Don’t know | 1  0  99 |  |

| **No.** | **Final observation and end** | **Answer Choices** | **Code** | **Logic** |
| --- | --- | --- | --- | --- |
| H1a | This concludes our survey. Thank you very much for your time.  *Give soap to respondent.* | | |  |
| GPS | TAKE GPS MEASUREMENT. *As close as possible to the household.* | | |  |
| H1b | WRITE HH ID. |  |  |  |
| H2a | ENUMERATOR: have you already inspected the surroundings of this household when surveying a previous household? | Yes  No  Don’t know | 1  0  99 | >>H3 |
| H2b | OBSERVE: Are there human feces present in the immediate environment of the household, outside a toilet?  *Check surroundings and refuse dump.* | Yes  No  Cannot observe | 1  0  99 |  |
| H3 | Any comments or notes from the enumerator.  *Indicate if you have reasons to believe that the respondent was not truthful.* |  |  |  |

| **ADDITIONAL QUESTIONS FOR ENDLINE SURVEY** | | | | | | |
| --- | --- | --- | --- | --- | --- | --- |
| **No.** | **Endline questions** | **Answer Choices** | **Code** | **Logic** | |  |
| I1 | Did you receive a toilet voucher? | Yes  No  Don’t know | 1  0  99 | >>I15  >>I15 | |  |
| I2 | Did you redeem your voucher? | Yes  No  Don’t know | 1  0  99 | >>I6  >> I15 | |  |
| I3 | Why did you not redeem your voucher?  *[Select all that apply]* | Did not want new toilet  Lost the voucher  Forgot about the voucher  Didn’t know how to redeem it  Household member refused to redeem it  Artisan refused to come  Could not dig pit  Could not build superstructure Sold the voucher  Gave away the voucher  Other: ___________  Don’t know | 1  2  3  4  5  6  7  8  9  10  96  99 | >> I15  >> I15  >> I15  >> I15  >> I15  >> I15  >> I15  >> I15  >> I15  >> I15 | |  |
| I4 | Who did you sell/give the voucher too? | Family member  Other community member  Someone outside community  Other: ___________  Don’t know | 1  2  3  69  99 |  | |  |
| I5 | How much did you sell/give the voucher for? | ____________ GHC |  | >>I15 | |  |
| I6 | Who dug the pit?  *[Select all that apply]* | Household  Other family members or neighbors  Artisan  Other: ____________  Don’t know | 1  2  3  96  99 |  | |  |
| I7 | Did you have to pay someone to dig the pit? | Yes  No  Don’t know | 1  0  99 | >>I8  >>I8 | |  |
| I8 | How much did you pay for the pit excavation? |  | | |  | |
| I9 | Who built the superstructure?  *[Select all that apply]* | Household  Other family members or neighbors  Artisan  Other: ____________  Don’t know | 1  2  3  96  99 | |  | |
| I10 | Did you have to purchase materials for the superstructure? | Yes  No  Don’t know | 1  0  99 | |  | |
| I11 | Did you have to pay someone for the superstructure? | Yes  No  Don’t know | 1  0  99 | |  | |
| I12 | *[If G8=1 or G9=1]*  In total, how much did you spend for the superstructure? |  | | |  | |
| I13 | Did your household give any money to the artisan who built the latrine? | Yes  No  Don’t know | 1  0  99 | | >> I15  >> I15 | |
| I14 | How much did you pay him approximately? |  | | |  | |
| I15 | Any comments or notes |  | | |  | |
