# Supplementary material for: Can open-defecation free (ODF) communities be sustained? A cross-sectional study in rural Ghana
Source: PLoS One. 2022 Jan 7;17(1):e0261674. doi: 10.1371/journal.pone.0261674 (PMC8740968; doi:10.1371/journal.pone.0261674)
Supplement: S2 Text — (DOCX) [file pone.0261674.s010.docx]

**WASHPaLS Research on the Impact of Targeted Subsidies within ODF Communities**

**Baseline Village survey**

| Geographic location | District: |  |
| --- | --- | --- |
|  | Town/Village: |  |
|  | GPS coordinates |  |
| Chief/Assistant Chief/Village elder | Chief name: |  |
|  | Chief gender: | Male/Female |

| No. | Question | Answer Choices | Code | Logic |
| --- | --- | --- | --- | --- |
| A1 | Is one of the chiefs or village elders who is >18 available to be interviewed? | Yes  No | 1  0 | >>End |
| A2 | READ CONSENT FORM  Are you willing to participate in the study? | Yes  Yes, though at a later time: _____  No | 1  2  0 | >>Return  >>End |
| A3 | How many times have you tried to interview the chief or village elder? | _________ |  | >>End if >2 |
| A4 | May the chief or a village elder who is ≥18 years old be available at a later time? | Yes  No | 1  0 | >>A_6  >>End |
| A5 | When would be a good time to return? | _____________ |  |  |
| A6 | Family name/last name: |  |  |  |
| A7 | Respondent first name: |  |  |  |
| A8 | Respondent popular name: |  |  |  |
| A9 | Respondent gender: | Female  Male | 2  1 |  |
| A10 | Respondent status: | Chief  Council of elder  Community elder  Assembly man  Unit committee member  Other: ___________ | 1  2  3  4  5  96 |  |
| **No.** | **Demographics** | **Answer Choices** | **Code** | **Logic** |
| B1 | How many houses does this community have? |  | |  |
| B2 | How many households does this community have? |  | |  |
| B3 | In your opinion, what are the main problems in this community?  *Select up to 3.* | Poverty  Lack of food  Lack of jobs  Education  Road access  Electricity  Flooding  Drought  Child health  Hospital access  Water supply  Sanitation  Other: _________  Don’t know | 1  2  3  4  5  6  7  8  9  10  11  12  96  99 |  |
| B4 | Are some community members enrolled in the LEAP program? (do they have a card that entitles them to financial help from the District Assembly?) | Yes  No  Don’t know | 1  0  99 |  |
| B5 | Does this community have any internal conflicts? | Yes  No  Don’t know | 1  0  99 |  |
| B6 | Does this community have a Village Savings and Loans Association? | Yes  No  Don’t know | 1  0  99 |  |
| **No.** | **Hydrogeology** | **Answer Choices** | **Code** | **Logic** |
| C1 | If you start digging, how far do you have to dig before you get water?  *If it depends on where you are in the village, select the shallowest depth.* | Less than 5 feet  5-10 feet  10-15 feet  More than 15 feet  Don’t know | 1  2  3  4  99 |  |
| C2 | What are the soil characteristics in this community (where people reside)?  *Select all that apply.*  *0 and 99 not compatible with other options* | Sandy  Rocky  Loam  Clay  Non-stable  Difficult to dig  None of the above  Other: ________  Don’t know | 1  2  3  4  5  6  0  96  99 |  |
| C3 | Does this village or parts of this village flood regularly? | Yes, every year  Yes, but less than every year  No  Don’t know | 1  2  0  99 |  |
| C4 | Is there a water body (lake, river, pond) nearby, i.e. within 5 min walk? | Yes  No  Don’t know | 1  0  99 |  |
| C5 | Is there dense vegetation (forest, dense bushes, high grasses) nearby, i.e. within 5 min walk? | Yes  No  Don’t know | 1  0  99 |  |
| **No.** | **Sanitation** | **Answer Choices** | **Code** | **Logic** |
| D1 | In this community, what proportion of households own a toilet? | All  Most  Approximately half  Some  None  Don’t know | 4  3  2  1  0  99 |  |
| D2 | In some communities, people use the bush or dig & burry to defecate. Do you know if that happens in this community? | Yes, regularly  Yes, but rarely  Yes, but only for children  No  Don’t know | 1  2  3  0  99 |  |
| D3 | Has this community received CLTS triggering? (i.e. been sensitized about toilets and sanitation?) | Yes  No  Don’t know | 1  0  99 |  |
| D8 | Has this community been verified ODF? | Yes  No  Don’t know | 1  0  99 | >>D4  >>D4 |
| D9 | In what year was this community verified ODF? | 2015  2016  2017  2018  2019  Don’t know | 15  16  17  18  19  99 |  |
| D4 | What happens if a community member is found open defecating? (are there any rules or by-laws that apply in this case?)  *Select all that apply* | Warning  Fine  Other: _______  None  Don’t know | 1  2  96  0  99 | >>D6  >>D6 |
| D5 | Have you taken these actions in the past year? | Yes  No  Don’t know | 1  0  99 |  |
| D6 | Does this community have any members trained on latrine construction?  *e.g., artisans, masons, community technical volunteers.* | Yes  No  Don’t know | 1  0  99 |  |
| D7 | Has this community received other NGO programs related to water or sanitation?  *Select all that apply* | Water supply  Sanitation  Handwashing  Other: _______  Don’t know  None | 1  2  3  96  99  0 |  |
| **No.** | **Water** | **Answer Choices** | **Code** | **Logic** |
| E1 | What are the main water sources for drinking and cooking in this community?  *Select all that apply* | Piped water  Borehole/tube-well  Protected dug well  Unprotected dug well  Protected spring  Unprotected spring  Rainwater  Surface water  Sachet water  Other: _______  Don’t know | 1  2  3  4  5  6  7  8  9  96  99 | >> F1 |
| E2 | For each source mentioned:  Where is this water source located?  *How long does it take to get there?* | Within community  Less than 10 min away  More than 10 min away  Don’t know | 1  2  3  99 |  |
| **No.** | **Final observation and end** | **Answer Choices** | **Code** | **Logic** |
| F1 | This concludes our survey. Would you like to add anything? |  |  |  |
| F2 | Any comments or notes from the supervisor. Indicate if you have reasons to believe that the respondent was not truthful. |  |  |  |
| F3 | SUPERVISOR: did you see the ODF certificate with the correct community name? | Did not see certificate  Saw certificate with correct community name  Saw certificate but community name was different: __________  Other: __________ | 0  1  2  96 |  |
| F4 | SUPERVISOR: were you able to verify that the contact given by the District Assembly lives in this community? | Yes, verified  No, could not verify  DA did not provide contact | 1  0  99 |  |
